# Lack of Association between Inhaled Corticosteroid Use and the Risk of Future Exacerbation in Patients with GOLD Group A Chronic Obstructive Pulmonary Disease

**DOI:** 10.3390/jpm12060916

**Published:** 2022-05-31

**Authors:** Sun Hye Shin, Deog Kyeom Kim, Sang-Heon Kim, Tae Rim Shin, Ki-Suck Jung, Kwang Ha Yoo, Ki-Eun Hwang, Hye Yun Park, Yong Suk Jo, on behalf of the KOCOSS Cohort

**Affiliations:** 1 Division of Pulmonary and Critical Care Medicine, Department of Medicine, Samsung Medical Center, Sungkyunkwan University School of Medicine, Seoul 06351, Korea; freshsunhye@gmail.com; 2 Department of Internal Medicine, Seoul National University College of Medicine, SMG-SNU Boramae Medical Center, Seoul 07061, Korea; kimdkmd@gmail.com; 3 Department of Internal Medicine, Hanyang University College of Medicine, Seoul 04763, Korea; sangheonkim@hanyang.ac.kr; 4 Lung Research Institute, Hallym University College of Medicine, Chuncheon 24252, Korea; trshinmd@hanmail.net (T.R.S.); pulmoks@hallym.ac.kr (K.-S.J.); 5 Division of Pulmonary, Allergy, and Critical Care Medicine, Department of Internal Medicine, Hallym University Kangnam Sacred Heart Hospital, Seoul 07441, Korea; 6 Department of Pulmonary, Allergy and Critical Care Medicine, Hallym University Sacred Heart Hospital, Anyang 14068, Korea; 7 Division of Pulmonary and Allergy, Department of Internal Medicine, Konkuk University School of Medicine, Seoul 05030, Korea; khyou@kuh.ac.kr; 8 Department of Internal Medicine, Wonkwang University School of Medicine, Iksan 54538, Korea; ke1205@hanmail.net; 9 Division of Pulmonary and Critical Care Medicine, Department of Internal Medicine, College of Medicine, Seoul St. Mary’s Hospital, The Catholic University of Korea, Seoul 06591, Korea

**Keywords:** COPD, exacerbation, inhaled corticosteroid, inhaler

## Abstract

Background: As most clinical trials have been performed in more symptomatic and higher-risk patients, evidence regarding treatment in patients with Global Initiative for Chronic Obstructive Lung Disease (GOLD) group A chronic obstructive pulmonary disease (COPD) is limited. We assessed the distribution of inhaler treatment and sought to investigate the association between inhaled corticosteroid (ICS) use and future exacerbation in GOLD group A COPD patients. Methods: Patients with GOLD group A COPD who received maintenance inhalers were identified from a multicentre, prospective cohort in South Korea. Patients were categorized as group A when they had fewer symptoms and did not experience severe exacerbation in the previous year. Development of moderate or severe exacerbation during the 1-year follow-up was analysed according to baseline inhaler treatment. Results: In 286 patients with GOLD group A COPD, mono-bronchodilator (37.8%), dual-bronchodilator (29.0%), triple therapy (17.5%), and ICS/long-acting beta-2 agonist (15.4%) were used. Compared to patients without ICS-containing inhalers (*N =* 191), those using ICS (*N =* 95) were more dyspnoeic, and more likely to have asthma history, lower lung function, and bronchodilator response. During the 1-year follow-up, moderate or severe exacerbations occurred in 66 of 286 (23.1%) patients. In the multivariable logistic regression analysis, ICS-containing inhaler use was not associated with the development of exacerbation, even in the subgroup with a high probability of asthma–COPD overlap. Conclusion: Although about one-third of patients with GOLD group A COPD were using ICS-containing inhalers, use of ICS was not associated with a reduction in the future development of exacerbation.

## 1. Introduction

Chronic obstructive pulmonary disease (COPD) is contributing to 3.2 million deaths worldwide each year, leading to the third leading cause of mortality [1]. During the clinical course of COPD, some COPD patients are prone to suffering from recurrent exacerbations, which are significantly associated with a rapid decline in lung function [2], poor quality of life [3], increased mortality [4], and substantial socioeconomic burden [5]. Thus, one of the main goals of COPD management is to reduce the risk of future exacerbations [6].

As there is a variable degree of symptoms and exacerbations despite the same degree of airflow limitation, the “ABCD” assessment tool for COPD, which was first proposed in 2011 [7] and revised in 2017 [8], combines symptom burden and history of exacerbation with future risk of exacerbation. Accordingly, COPD patients were grouped into four categories (groups A to D), and group A was classified as having low risk for exacerbation and a less symptomatic group. The 2017 Global Initiative for Chronic Obstructive Lung Disease (GOLD) reports recommend a bronchodilator therapy (either short- or long-acting bronchodilator) in group A patients and suggest the initiation of long-acting bronchodilators, as either long-acting muscarinic receptor antagonists (LAMAs) or long-acting beta-2 agonists (LABAs), as maintenance treatment. However, evidence for this recommendation in group A is scarce because most clinical trials have been conducted in more symptomatic patients or those with a previous history of exacerbation (i.e., higher-risk patients) [9,10,11,12,13].

Furthermore, inhaled corticosteroids (ICS)-containing inhalers remain widely overused even in group A patients in real-world clinical practice [14]. However, there are no available data on the ICS effect on reducing exacerbations in Group A COPD patients. Given that group A patients account for approximately three-quarters of the COPD population [15,16,17], it is imperative to investigate the risk of future exacerbation based on the ICS use in this group. Thus, we aimed to assess the pattern of inhaler treatment and examine the association between the ICS use and risk of future exacerbation in patients with GOLD A COPD in real practice.

## 2. Materials and Methods

### 2.1. Study Population

We retrospectively identified participants from the Korean COPD subgroup study (KOCOSS) cohort (registered on ClinicalTrials.gov with identifier CT02800499), an ongoing multicentre prospective cohort for investigating the COPD phenotype of patients in the Republic of Korea, the details of which have been published previously [18]. Briefly, eligible patients were 40 years or older and received a diagnosis of COPD by spirometry with confirmed fixed airflow obstruction (post-bronchodilator [BD] forced expiratory volume in 1 s [FEV_1_] /forced vital capacity [FVC] ratio of <0.7). For the present study, we included patients with GOLD group A enrolled between 1 January 2012 and 31 December 2019. Patients in GOLD group A were defined as those having modified Medical Research Council (mMRC) < 2 and COPD assessment test (CAT) < 10 and those who did not experience severe exacerbations requiring hospitalization during the year prior to enrolment. Patients were excluded from the analysis when they were followed up for less than a year, or there were no data regarding exacerbation during the follow-up period. Patients who did not receive maintenance inhaler therapy were also excluded from the analysis.

### 2.2. Measurement of Clinical Data

At enrolment, data including age, sex, smoking history, pack years, comorbidities, symptoms (based on questionnaires including the mMRC), and health-related quality of life (based on the CAT and the St. George’s Respiratory Questionnaire [SGRQ]) were collected by physicians or trained nurses using electronic case report forms.

Pulmonary function tests were performed using standard equipment in accordance with the American Thoracic Society/European Respiratory Society guidelines [19,20]. The predicted percentage values were calculated using an equation developed in the Korean population [21,22]. Bronchodilator response (BDR) positivity was defined as elevated FEV_1_ >12% and >200 mL from baseline FEV_1_ after the inhalation of 200 µg of salbutamol [23]. Exercise capacity was measured using a 6 min walking distance (6 MWD). Blood samples were collected from patients for baseline laboratory evaluations, including eosinophil counts, during a stable period.

The presence of asthma–COPD overlap (ACO) was defined as the presence of either (a) an improvement in FEV_1_ greater than 400 mL and greater than 15% following bronchodilator administration and/or (b) blood eosinophil count ≥300 cells/μL, which is applicable in our cohort based on the most recently proposed diagnostic criteria for ACO [24].

The use of medications, including LAMA, LABA, and/or ICSs, was recorded at enrolment and at each visit at 6-month intervals. The exposure in this study was maintenance inhaler medication at baseline, which was divided into monotherapy (LAMA or LABA alone), dual-bronchodilator therapy (LAMA/LABA combined), ICS/LABA, and triple therapy (ICS/LAMA/LABA combined) and then categorized into ICS-containing inhalers and non-ICS-containing inhalers.

### 2.3. Outcome Measure

The risk of moderate or severe exacerbation within 1 year after enrolment was analysed. Patients were followed-up at least every 6 months, and exacerbation data were recorded at every visit. Exacerbation was assessed using the question “Have you had an aggravation of your respiratory symptoms that led to a visit to the outpatient clinic sooner than planned, hospitalization, or a visit to the emergency department during the past 6 months?” Moderate exacerbation was defined as an exacerbation that led to a visit to an outpatient clinic earlier than scheduled with a prescription of systemic steroids and/or antibiotics, whereas severe exacerbation was defined as an exacerbation that led to a visit to the emergency department or hospitalization.

### 2.4. Ethics

All hospitals involved in the study obtained approval from their respective institutional review board committees and informed consent was obtained from their patients. The study protocol was approved by the Institutional Review Board of the KONKUK University Medical Center (IRB No. KHH1010338).

### 2.5. Statistical Analysis

All data are presented as numbers (%) for categorical variables and mean (standard deviation, SD) for continuous variables. Categorical variables were compared using the Pearson chi-square test or Fisher’s exact test, while Student’s *t*-test was used to compare continuous variables. The risk of exacerbation during the 1-year follow-up period was analysed using logistic regression analysis. Covariates including age, sex, smoking history, body mass index (BMI), post-bronchodilator FEV_1_ at baseline, history of moderate exacerbation during the year prior to enrolment, and high probability of ACO were adjusted in the multivariable analysis. Subgroup analysis based on possible confounders for ICS use (past history of moderate exacerbation, blood eosinophils, history of asthma, and lower lung function) was performed. To exclude the effects of patients with a high likelihood of ACO, patients with a high probability of ACO and those with missing blood eosinophil data were excluded and reanalysed. All tests were two-sided, and a *p*-value < 0.05 was considered statistically significant. All analyses were performed using STATA software (ver. 14.2; StataCorp, College Station, TX, USA).

## 3. Results

### 3.1. Description of Participants

**Figure 1:**
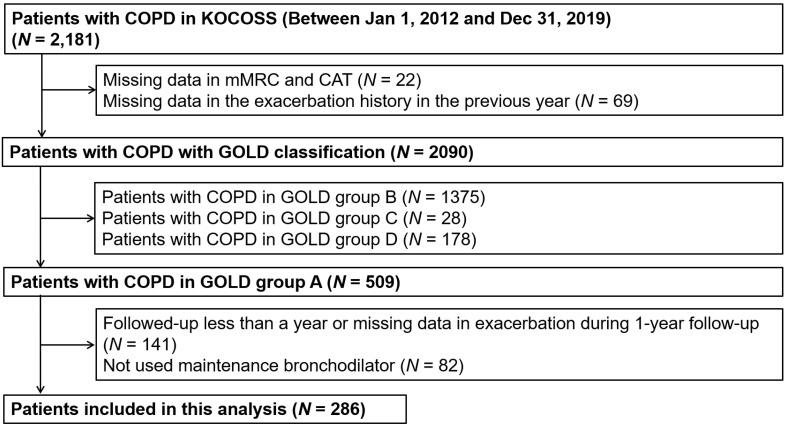
Selection of subjects. COPD: chronic obstructive pulmonary disease; GOLD: Global Initiative for Chronic Obstructive Lung Disease.

Among the patients with COPD from the KOCOSS who could be classified into GOLD group categories (*N =* 2090), the largest group was group B (66%), followed by group A (24%), group D (9%), and group C (1%; Figure S1). This study only included patients in group A, who were receiving maintenance inhaler therapy and had exacerbation data during the 1-year follow-up. Finally, 286 patients were included in the analysis (Figure 1).

As presented in Table 1, the mean age was 68.3 years, and most patients were male and former or current smokers. Corresponding to the definition of GOLD group A, the mean (SD) mMRC, CAT, and total SGRQ scores were 0.66 (0.48), 5.8 (2.3), and 16.4 (9.6), respectively. In addition, none of the patients experienced severe exacerbation during the previous year, and only 24 (8.4%) patients experienced any (at least one) moderate exacerbation during the previous year.

### 3.2. Inhaler Prescription Status

**Figure 2:**
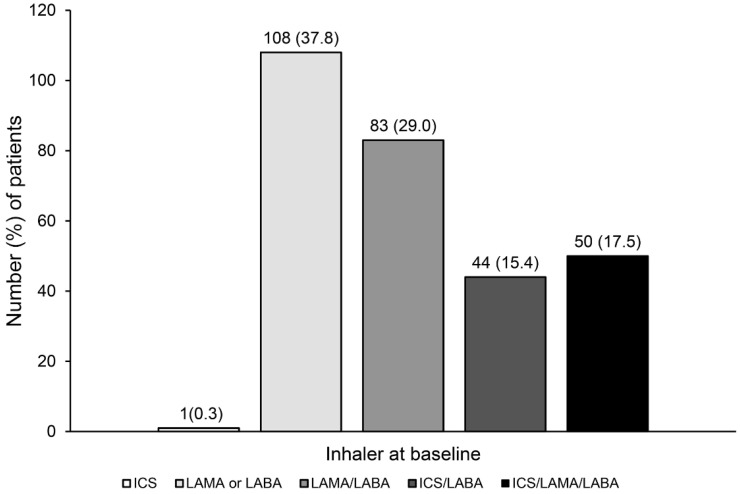
Prescription status of inhaler treatment in GOLD A COPD subjects. CAT: chronic obstructive pulmonary disease assessment test; COPD, chronic obstructive pulmonary disease; GOLD, Global Initiative for Chronic Obstructive Lung Disease; ICS, inhaled corticosteroid; LABA, long-acting beta2-agonist; LAMA, long-acting muscarinic antagonist.

Regarding the maintenance inhaler treatment at baseline, LAMA or LABA monotherapy was the most frequently administered (37.8%), followed by dual-bronchodilator therapy (29.0%), triple therapy (17.5%), and ICS/LABA therapy (15.4%) (Figure 2). Approximately one-third (*N =* 95) of COPD patients in GOLD group A were prescribed ICS-containing inhalers.

### 3.3. Clinical Characteristics According to ICS Use

Compared with patients without ICS-containing inhalers (*N =* 191), those using ICS (*N =* 95) were more dyspnoeic (patients with mMRC 1, 61.8% vs. 73.7%, *p* = 0.046), more likely to have asthma history (31.4% vs. 45.3%, *p* = 0.046), lower FEV_1_ (% pred, 64.0 vs. 58.6, *p* = 0.005), lower FEV_1_/FVC ratio (0.54 vs. 0.51, *p* = 0.012), and positive BDR (4.7% vs. 14.7%, *p* = 0.003). However, there was no difference in the history of moderate exacerbation in the previous year, blood eosinophil count, or ACO between the ICS and non-ICS groups. Additionally, the number of bronchodilators used was not different between two groups (Figure S2).

### 3.4. Development of Exacerbation According ICS Use

Moderate to severe exacerbation occurred in 66 of 286 (23.1%) patients during the 1-year follow-up period (41 of 191 [21.5%] in patients without ICS vs. 25 of 95 [26.3%] in patients with ICS, *p* = 0.359). In the multivariable logistic regression model, ICS-containing inhaler use was not associated with the development of exacerbation (Table 2). A subgroup analysis of clinical features (past history of exacerbation, blood eosinophil count, previous asthma history, and low FEV_1_) that might confound the ICS treatment was performed. In all subgroups, ICS-containing inhaler therapy was not associated with the risk of exacerbation, and the interactions were not significant (Table 3).

### 3.5. Development of Exacerbation According the Type of Inhaler Therapy

When the analysis was conducted in four groups based on the type of inhaler therapy, moderate to severe exacerbation occurred in 23.2% (25 of 108) in the monotherapy group, 19.3% (16 of 83) in the dual-bronchodilator therapy group, 25% (11 of 44) in the ICS/LABA group, and 26% (13 of 50) in the triple therapy group. In multivariable adjusted models with the monotherapy group as a reference, dual-bronchodilator, ICS/LABA, and triple therapy did not significantly affect the risk of exacerbation (Table 4). After excluding patients with a high probability of ACO (*N =* 44) and those with missing blood eosinophils (*N =* 59), similar results were observed (Table S1).

## 4. Discussion

In this study, using data from a multicentre cohort of patients with COPD, we found that various inhaler treatments from mono-bronchodilator to triple therapy were used in GOLD group A patients, who are, by definition, less symptomatic and low-risk. Unlike recommendations from the current clinical practice guidelines, approximately one-third of these group A COPD patients received ICS-containing inhaler therapy in real practice. Of note, moderate-to-severe exacerbations occurred in 23.1% of patients during the 1-year follow-up. However, ICS-containing inhaler therapy was not associated with a reduced risk of exacerbations, which persisted even in patients with Th2 inflammatory clinical features.

Although inhaled bronchodilator therapy is the pivotal treatment for stable COPD, evidence regarding treatment for GOLD group A patients is very limited. In general, approximately 75% of COPD patients remain undiagnosed, and most of them are less symptomatic [25,26]. These patients rarely receive treatments or make regular visits to the hospital, being largely under-represented in most clinical trials and clinic-based cohort studies. However, a general-population-based study showed that undiagnosed “asymptomatic” COPD patients are at higher risk of exacerbations and mortality, contributing to a significant burden on the health care system [25,27]. Indeed, LAMA in COPD patients with mild airflow limitation (GOLD 1 or 2) or younger COPD patients showed a favourable effect not only on lung function decline but also on reduction in exacerbation [12,28]. Moreover, current guidelines recommend a bronchodilator either short- or long-acting in GOLD A with low-risk and less symptomatic patients. Nevertheless, our study focusing on the GOLD A group showed that 33% were prescribed ICS-containing inhalers, and half of them used triple inhalers. It is well-known that over-use of ICS in combination with long-acting beta-agonists is widely used in the management of COPD in real practice, regardless of disease severity [29,30,31]. In a nationwide database of South Korea, ICS-containing inhalers were prescribed in about 50% of COPD patients over 3 years through an assessment of adequacy for COPD care [32]. We again showed the over-use of ICS even in COPD patients with less symptoms and a low risk for exacerbation. There are several possible reasons behind the overuse of ICS in this GOLD group A patients. In our study, those using ICS at baseline were more dyspneic and had a lower lung function. It is well-known that a higher mMRC grade at baseline was predictive of future exacerbation [33]. Thus, it is possible that ICS-containing inhalers were prescribed in more dyspneic patients to reduce not only symptoms but also future exacerbation risk. Another characteristic of ICS users in our study was that they were more likely to have asthma history and positive BDR, compared with non-ICS users. These two factors are also known to be associated with higher exacerbation risk [34,35] and might have affected the ICS use in GOLD group A patients. However, ICS use did not show a benefit in low-risk/less symptomatic COPD patients. Accordingly, concerns have been raised about the overuse of ICS as maintenance therapy in relation to pneumonia and other adverse effects from long-term use of ICS [36,37].

Regarding disease burden such as exacerbations, we found that quite a number of exacerbations were still developing in group A (23.1%) during the following year. Moreover, we showed that the risk of exacerbation was similar across the various types of inhaler therapy used in this group of patients; most importantly, ICS use was not associated with a reduction in exacerbation risk in this group. Current guidelines recommend the use of ICS in combination with long-acting bronchodilators as an initial treatment only for COPD patients who have exacerbation risk and exhibited clinical features of Th2-cell signature including elevated blood eosinophil count [38,39], and as a follow-up add-on treatment for those who experience frequent exacerbation (≥2 moderate exacerbation) or more than one severe exacerbation, based on large-scale clinical trials [6]. Recent clinical trials on triple therapy showed the benefit of triple therapy over LAMA/LABA in preventing acute exacerbation [40,41,42] and even in reducing mortality [43,44]. However, these survival benefits are likely to be specific to a high-risk population with frequent exacerbation, as a recent pooled analysis of over 6000 patients with mild-to-very-severe COPD and low exacerbation risk showed no difference in time to death [45]. Likewise, the difference in ICS benefit in exacerbation prevention between our study and the recent clinical trials on triple therapy is due to the marked difference in the patient characteristics. Recent clinical trials of triple therapy included symptomatic (CAT ≥ 10) patients, with at least one moderate or one severe exacerbation (in those FEV1 ≥ 50%) or two moderate or one severe exacerbation (in those FEV1 < 50%) [40,41,42]. In contrast, our study population were far less symptomatic (mean CAT score 5.8) and only 8.4% had history of moderate exacerbation. None had history of severe exacerbation. Thus, our study supports the current GOLD recommendations that ICS should be reserved for the high-risk patients and those with eosinophilic phenotype.

Elevated peripheral blood eosinophil counts in COPD may be used to identify patients who are expected to have a favourable response to ICS therapy or targeting agents for the Th2 inflammatory pathway. Elevated blood eosinophil level was associated with increased exacerbation risk and this group of patients could benefit from corticosteroid therapy directed against eosinophilic pathway [40,46,47,48]. In this study, blood eosinophil count was not different between ICS users and non-ICS users (231.3 ± 247.7 cells/μL vs. 204.4 ± 205.6 cells/μL, *p* = 0.386) and ICS use did not reduce exacerbation risk in group A COPD patients. Thus, we conducted a subgroup analysis based on these indications of ICS use and showed that the use of ICSs did not reduce the risk of exacerbation in the high blood eosinophil count (≥300 cells/μL) group, a commonly used biomarker for Th2 inflammation, nor in groups with past history of moderate exacerbations, and self-reported asthma. Even when we adjusted for high probability of ACO (the extreme BDR and high blood eosinophil count according to the most recently proposed diagnostic criteria for ACO [24]), there was no significant reduction in exacerbation according to ICS use. Moreover, the same results were found in the analysis that excluded patients with a high ACO potential (Table S1). The use of ICS, which was not based on reliable biomarkers for beneficial response to ICS, in low-risk and less symptomatic group A patients, did not improve outcome. Taken together, these results suggest that overuse of ICS has no benefit in such low-risk/less symptomatic COPD patients, even when accompanied by clinical features implying Th2 inflammation. Thus, our results support the current practice guidelines that do not recommend ICS use in patients with GOLD group A COPD and suggest that mono-bronchodilator therapy alone (without ICS) might be good enough for exacerbation prevention in this low-risk group [49]. Rather, the goal of treatment in this group should be more focused on symptoms or quality of life, and prevention of lung function decline.

This study has several limitations. First, there were only 8.4% of patients in our study population who had history of any moderate exacerbation in the prior year, which is a low number of at-risk patients to study with for exacerbation as the outcome. While this is in concordance with the definition of the study population of GOLD group A, the prevalence of previous exacerbation seems lower than those of previous studies (ranged from 14.4 to 20.9%) [50,51]. This might be due to selection bias, as we only included patients who were already being managed with maintenance inhalers at referral hospitals and thus having lower exacerbation in the previous year. Additionally, this might be attributable to recall bias, as the previous exacerbations were retrospectively collected at enrolment. Likewise, during the 1-year follow-up, the cumulative incidence of moderate acute exacerbation (19.9%) showed a more than 2-fold increase compared to baseline. This might reflect the increase in the case finding during the prospective data collection. Second, because patients in our study were recruited from outpatient clinics at tertiary referral hospitals in Korea, not from primary care clinics, the number of GOLD Group A patients was low and the results may not be representative of the general COPD population. Indeed, the prevalence of COPD in South Korea ranged from 13.1% to 14.6% between 2010 and 2015. However, only less than 5% visited hospitals for the management of COPD [52]. We speculate that most of the GOLD Group A patients remain undiagnosed as they are asymptomatic or mildly symptomatic, and rarely visit clinics. Third, as the KOCOSS cohort was not originally designed to identify a beneficial inhaler treatment to reduce future exacerbations, the analysis (by definition) had to be retrospective in nature. Accordingly, COPD patients in our study were enrolled irrespective of the treatment-naïve status.

## 5. Conclusions

A significant number of group A COPD patients were prescribed ICS-containing inhaler therapy in real-world clinical practice, even in cases where ICS is not recommended according to current guidelines. However, ICS use in group A COPD patients had no significant effect on reducing the risk of exacerbation compared to LAMA or LABA monotherapy.

## Figures and Tables

**Table 1:** Baseline characteristics of subjects.

	Overall (*N* = 286)	Without ICS (*N* = 191)	With ICS (*N* = 95)	*p* Value
Age (years)	68.3 ± 7.4	68.2 ± 7.5	68.5 ± 7.3	0.701
Male sex	269 (94.1)	179 (93.7)	90 (94.7)	0.731
Smoking (*N =* 287)				0.302
Never	29 (10.2)	17 (9.0)	12 (12.6)	
Former smoker	194 (68.1)	135 (71.1)	59 (62.1)	
Current smoker	62 (21.7)	38 (20.0)	24 (25.3)	
Pack years (*N =* 245)	42.1 ± 23.2	42.0 ± 23.6	42.2 ± 22.6	0.956
BMI (kg/m^2^)	23.5 ± 3.3	23.4 ± 3.4	23.6 ± 3.2	0.700
Education (above high school) (*N =* 284)	52 (18.3)	36 (18.9)	16 (17.2)	0.737
mMRC dyspnoea scale				0.046
0	98 (34.3)	73 (38.2)	25 (26.3)	
1	188 (65.7)	118 (61.8)	70 (73.7)	
Quality of life				
SGRQ score	16.4 ± 9.6	15.8 ± 9.2	17.8 ± 10.4	0.091
CAT score	5.8 ± 2.3	5.8 ± 2.4	5.7 ± 2.3	0.744
Moderate exacerbation in the prior year	24 (8.4)	16 (8.4)	8 (8.4)	0.990
Comorbidities				
Hypertension	111 (38.8)	74 (38.7)	37 (39.0)	0.973
Congestive heart failure	5 (1.8)	5 (2.6)	0 (0)	0.112
Ischemic heart disease	16 (5.6)	11 (5.8)	5 (5.3)	0.864
Dyslipidaemia	46 (16.1)	30 (15.7)	16 (16.8)	0.350
Diabetes mellitus	52 (18.2)	33 (17.3)	19 (20.0)	0.574
Gastro-oesophageal reflux	36 (12.6)	26 (13.6)	10 (10.5)	0.459
Osteoporosis	6 (2.1)	6 (3.1)	0 (0)	0.168
Tuberculosis	73 (25.5)	53 (27.8)	20 (21.1)	0.221
Asthma	103 (36.0)	60 (31.4)	43 (45.3)	0.022
Asthma–COPD overlap * (*N =* 229)	44 (19.2)	26 (17.0)	18 (23.7)	0.226
Spirometry				
Post-BD FVC, L	3.50 ± 0.76	3.54 ± 0.75	3.33 ± 0.77	0.025
Post-BD FVC, %predicted	83.5 ± 15.5	84.4 ± 14.8	81.7 ± 16.7	0.164
Post-BD FEV_1_, L	1.84 ± 0.51	1.91 ± 0.51	1.69 ± 0.47	<0.001
Post-BD FEV_1_, %predicted	62.2 ± 15.2	64.0 ± 15.2	58.6 ± 14.6	0.005
Post-BD FEV_1_/FVC	53.3 ± 10.6	54.4 ± 10.7	51.0 ± 10.0	0.012
BDR positivity ^†^	23 (8.0)	9 (4.7)	14 (14.7)	0.003
DLco, %predicted (*N* *=* 226)	70.5 ± 20.8	70.0 ± 21.9	71.4 ± 18.5	0.631
Exercise capacity, 6MWD (m) (*N* *=* 223)	425.2 ± 120.5	426.6 ± 113.6	422.2 ± 135.1	0.800
Blood eosinophil count (*N* *=* 229)	213.3 ± 220.3	204.4 ± 205.6	231.3 ± 247.7	0.386

* Asthma–COPD overlap is defined if there is either (a) an improvement in FEV_1_ greater than 400 mL and greater than 15% following bronchodilator administration and/or (b) blood eosinophil count ≥300 cells/μL. ^†^ BDR positivity was defined as elevated FEV_1_ >12% and >200 mL from baseline FEV_1_ after the inhalation of 200 µg of salbutamol. BD: bronchodilator, BDR: bronchodilator response; BMI: body mass index; CAT: chronic obstructive pulmonary disease assessment test; COPD: chronic obstructive pulmonary disease; DLco: diffusing capacity for carbon monoxide; FEV_1_: forced expiratory volume in 1 s; FVC: forced vital capacity; ICS, inhaled corticosteroid; mMRC: modified Medical Research Council; SGRQ: St. George’s Respiratory Disease Questionnaire; 6 MWD: 6 min walk distance.

**Table 2:** Risk of moderate or severe exacerbation according to ICS use.

	No (%) of Patients with Moderate or Severe Exacerbation during 1-Year Follow-Up Period	Odds Ratio (95% Confidence Interval)			
		Crude	Model 1	Model 2	Model 3
Without ICS (*N =* 191)	41 (21.5)	Reference	Reference	Reference	Reference
With ICS (*N* *=* 95)	25 (26.3)	1.31 (0.74–2.32)	1.22 (0.66–2.26)	1.24 (0.67–2.31)	1.37 (0.67–2.78)

Model 1: adjusted for age, sex, smoking (never, ex-, current), BMI, mMRC grade, CAT score, and post-bronchodilator FEV_1_ %predicted. Model 2: further adjusted for past exacerbation history to Model 1. Model 3: further adjusted for the high possibility of asthma–COPD overlap to Model 2.BMI: body mass index; CAT: chronic obstructive pulmonary disease assessment test; COPD: chronic obstructive pulmonary disease; FEV_1_: forced expiratory volume in 1 s; ICS: inhaled corticosteroid; mMRC: modified Medical Research Council.

**Table 3:** Subgroup analysis of the risk of moderate or severe exacerbation according to ICS use.

	No (%) of Patients with Moderate or Severe Exacerbation during 1-Year Follow-Up Period		Odds Ratio (95% Confidence Interval)		
			Crude	Model 1	*p* for Interaction
	Without ICS	With ICS			
Overall (*N =* 286)	41/191 (21.5)	25/95 (26.3)	1.31 (0.74–2.32)	1.22 (0.66–2.26)	
Past history of moderate AE					0.100
No (*N =* 262)	33/175 (18.9)	23/87 (26.4)	1.55 (0.84–2.84)	1.53 (0.80–2.94)	
Yes (*N =* 24)	8/16 (50.0)	2/8 (25.0)	0.33 (0.05–2.18)	0.36 (0.02–5.35)	
Blood eosinophil count (*N =* 229)					0.501
<300 cells/μL (*N =* 187)	23/127 (18.1)	16/60 (26.7)	1.64 (0.79–3.41)	1.52 (0.68–3.43)	
≥300 cells/μL (*N =* 42)	6/26 (23.1)	4/16 (25.0)	1.11 (0.26–4.75)	1.04 (0.17–6.49)	
Self-reported asthma					0.901
No (*N =* 183)	25/131(19.1)	13/52 (25.0)	1.41 (0.66–3.03)	1.38 (0.60–3.16)	
Yes (*N =* 103)	16/60 (26.7)	12/43 (27.9)	1.06 (0.44–2.56)	1.27 (0.45–3.58)	
Postbronchodilator FEV_1_, %predicted *					0.037
≥50% (*N =* 225)	32/158 (20.3)	14/67 (20.9)	1.04 (0.51–2.11)	1.11 (0.53–2.32)	
<50% (*N =* 61)	9/33 (27.3)	11/28 (39.3)	1.73 (0.59–5.07)	1.98 (0.60–6.58)	

Odds ratios were analysed by non-ICS use as reference. Model 1: adjusted for age, sex, smoking (never, ex-, current), BMI, mMRC grade, CAT score, and post-bronchodilator FEV_1_ %predicted. * For the subgroup analysis by post-bronchodilator FEV_1_ % pred (≥50% vs. < 50%), post-bronchodilator FEV_1_ %predicted was not adjusted in Model 1. AE: acute exacerbation; BMI: body mass index; CAT: chronic obstructive pulmonary disease assessment test; COPD: chronic obstructive pulmonary disease; FEV_1_: forced expiratory volume in 1 s; ICS: inhaled corticosteroid; mMRC: modified Medical Research Council.

**Table 4:** The risk of moderate or severe exacerbation in group A COPD patients using mono-bronchodilator therapy as a reference.

	No (%) of Patients with Moderate or Severe Exacerbation during 1-Year Follow-Up Period	Odds Ratio (95% Confidence Interval)			
		Crude	Model 1	Model 2	Model 3
Mono bronchodilator (*N =* 108)	25 (23.2)	Ref	Ref	Ref	Ref
Dual bronchodilator (*N =* 83)	16 (19.3)	0.79 (0.39–1.60)	0.59 (0.27–1.28)	0.62 (0.29–1.35)	0.60 (0.24–1.47)
ICS/LABA (*N =* 44)	11 (25.0)	1.11 (0.49–2.50)	1.03 (0.43–2.43)	1.11 (0.47–2.65)	1.29 (0.49–3.40)
Triple therapy (*N =* 50)	13 (26.0)	1.17 (0.54–2.53)	0.85 (0.36–2.02)	0.85 (0.35–2.04)	0.80 (0.28–2.27)

Model 1: adjusted for age, sex, smoking (never, ex-, current), BMI, mMRC grade, CAT score, and post-bronchodilator FEV_1_ %predicted. Model 2: further adjusted for past exacerbation history to Model 1. Model 3: further adjusted for the high possibility of asthma–COPD overlap to Model 2. BMI: body mass index; CAT: CAT: chronic obstructive pulmonary disease assessment test; COPD: chronic obstructive pulmonary disease; FEV_1_: forced expiratory volume in 1 s; GOLD: Global Initiative for Chronic Obstructive Lung Disease; ICS/LABA: inhaled corticosteroid/long-acting beta2-agonist; mMRC: modified Medical Research Council.

## Data Availability

The datasets used for the current study are available from the corresponding author upon reasonable request.

## Supplementary Materials

The following are available online at https://www.mdpi.com/article/10.3390/jpm12060916/s1. Table S1: The risk of moderate or severe exacerbation in group A COPD patients after excluding those with high probability of having asthma–COPD overlap (*N* = 184). Figure S1: Distribution of GOLD group categories of the patients with COPD from KOCOSS (*N* = 2090). Figure S2: Prescription status of inhaler treatment in GOLD A COPD subjects according to ICS use.

## Abbreviations

6MWD: 6 min walk distance; ACO: asthma–chronic obstructive pulmonary disorder overlap; BDR: bronchodilator response; BMI: body mass index; CAT: chronic obstructive pulmonary disease assessment test; COPD: chronic obstructive pulmonary disease; FEV_1_: forced expiratory volume in 1 s; FVC: forced vital capacity; GOLD: Global Initiative for Chronic Obstructive Lung Disease; ICS: inhaled corticosteroid; LABA: long-acting beta2-agonist; LAMA: long-acting muscarinic antagonist; mMRC: modified Medical Research Council; SGRQ: St. George’s Respiratory Disease Questionnaire.
