# Supplementary material for: Lack of Association between Inhaled Corticosteroid Use and the Risk of Future Exacerbation in Patients with GOLD Group A Chronic Obstructive Pulmonary Disease
Source: J Pers Med. 2022 May 31;12(6):916. doi: 10.3390/jpm12060916 (PMC9224662; doi:10.3390/jpm12060916)
Supplement: Supplementary file 1 [file jpm-12-00916-s001.zip › jpm-1719983-supplementary.pdf]

Supplementary Materials

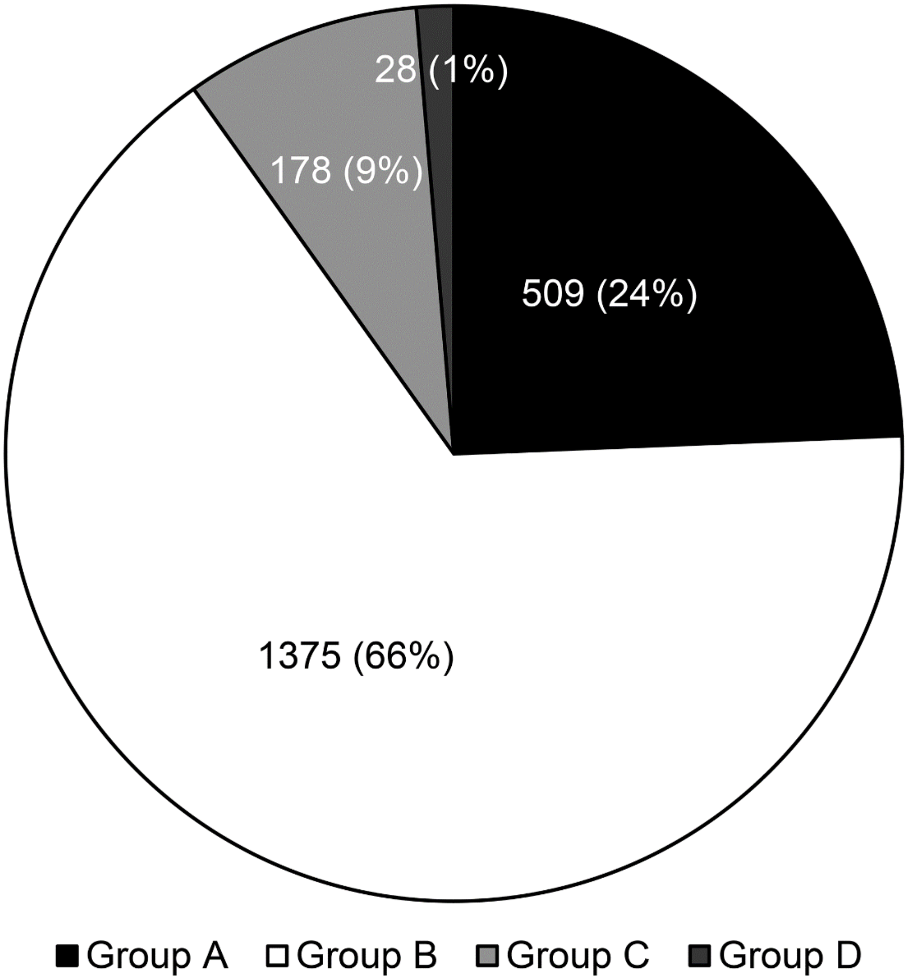

Figure S1. Distribution of GOLD group categories of the patients with COPD from KOCOSS (N = 2090). COPD, chronic obstructive pulmonary disease; GOLD, Global Initiative for Chronic Obstructive Lung Disease; KOCOSS, Korean COPD subgroup study.

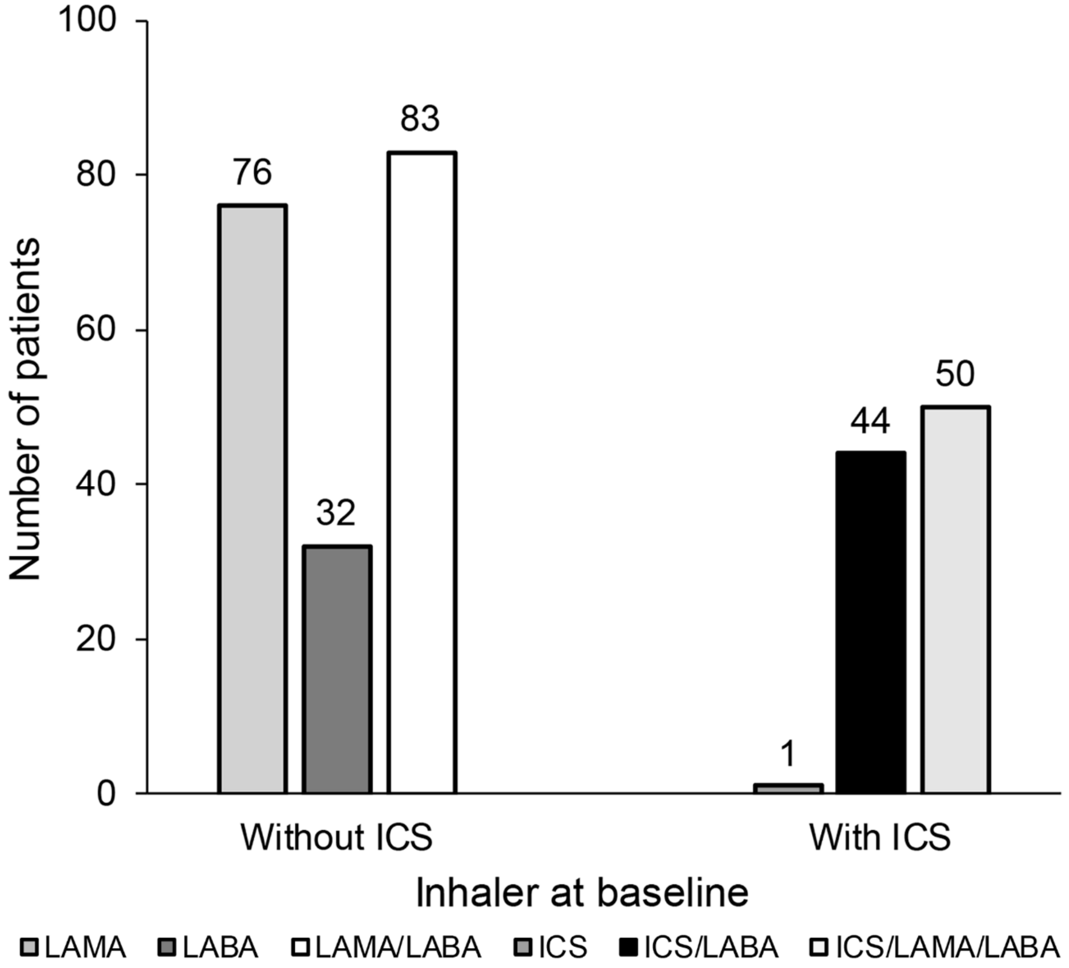

Figure S2. Prescription status of inhaler treatment in GOLD A COPD subjects according to ICS use. COPD, chronic obstructive pulmonary disease; GOLD, Global Initiative for Chronic Obstructive Lung Disease; ICS, inhaled corticosteroid; LABA, long-acting beta2-agonist; LAMA, long-acting muscarinic antagonist.

Table S1. The risk of moderate or severe exacerbation in group A COPD patients after excluding those with high probability of having asthma–COPD overlap (N = 184)

	No (%) of patients with moderated or severe exacerbation during 1-year follow-up period	Odds ratio (95% confidence interval)		
		Crude	Model 1	Model 2
Mono-bronchodilator (N = 67)	13 (19.4)	Ref	Ref	Ref
Dual-bronchodilator (N = 60)	10 (16.7)	0.83 (0.33–2.06)	0.58 (0.21–1.57)	0.58 (0.21–1.61)
ICS/LABA (N = 29)	9 (31.0)	1.87 (0.69–5.04)	1.83 (0.61–5.49)	1.92 (0.64–5.77)
Triple therapy (N = 28)	6 (21.4)	1.13 (0.38–3.36)	0.63 (0.18–2.24)	0.60 (0.16–2.20)

Model 1: adjusted for age, sex, smoking (never, ex-, current), BMI, mMRC grade, CAT score, and post-bronchodilator FEV_1 %predicted.

Model 2: further adjusted for past exacerbation history to Model 1.

BMI: body mass index; CAT: CAT: chronic obstructive pulmonary disease assessment test; COPD: chronic obstructive pulmonary disease; FEV_1: forced expiratory volume in 1 s; GOLD: Global Initiative for Chronic Obstructive Lung Disease; ICS/LABA: inhaled corticosteroid/long-acting beta2-agonist; mMRC: modified Medical Research Council.
